# A phase 1/2 study of NS-87/CPX-351 (cytarabine and daunorubicin liposome) in Japanese patients with high-risk acute myeloid leukemia

**DOI:** 10.1007/s12185-024-03733-z

**Published:** 2024-03-26

**Authors:** Kensuke Usuki, Toshihiro Miyamoto, Takuji Yamauchi, Kiyoshi Ando, Yoshiaki Ogawa, Masahiro Onozawa, Takahiro Yamauchi, Hitoshi Kiyoi, Akira Yokota, Takayuki Ikezoe, Yuna Katsuoka, Satoru Takada, Nobuyuki Aotsuka, Yasuyoshi Morita, Takayuki Ishikawa, Noboru Asada, Shuichi Ota, Atsushi Dohi, Kensaku Morimoto, Shunji Imai, Umi Kishimoto, Koichi Akashi, Yasushi Miyazaki, The Study Group for NS-87/CPX-351, Junya Kuroda, Hiroatsu Iida, Naohiro Sekiguchi, Katsuto Takenaka, Toshiro Kawakita, Kazunori Imada, Takahiro Suzuki, Shuichi Miyawaki, Noriko Usui, Norio Asou, Masakazu Muta, Kazuto Tsuruda, Masafumi Taniwaki, Masatoshi Fujita, Hideki Makishima, Yoko Nakanishi, Masaya Tajima, Yutaka Masutomi, Masahiro Chiba, Mayuna Hokazomo, Shihomi Hirooka, Taisuke Mikasa, Moemi Okamoto, Akitaka Kawase, Akane Yamada, Yuto Shimizu, Kento Isogaya, Tomohiko Ichikawa

**Affiliations:** 1 Department of Hematology, NTT Medical Center Tokyo, 5-9-22 Higashi-Gotanda, Shinagawa-Ku, Tokyo, 141-8625 Japan; 2 Department of Hematology, Institute of Medical Pharmaceutical and Health Sciences, Kanazawa University, Kanazawa, Ishikawa Japan; 3 Department of Medicine and Biosystemic Science, Graduate School of Medical Sciences, Kyusyu University, Fukuoka, Japan; 4 Department of Hematology and Onclogy, Tokai University School of Medicine, Isehara, Kanagawa Japan; 5 Department of Hematology, Hiroshima University School of Medicine, Hiroshima, Japan; 6 Department of Hematology, Hokkaido University Hospital, Sapporo, Hokkaido Japan; 7 Department of Hematology and Oncology, University of Fukui, Fukui, Japan; 8 Department of Hematology and Oncology, Nagoya University Graduate School of Medicine, Nagoya, Aichi Japan; 9 Department of Hematology, Chiba Aoba Municipal Hospital, Chiba, Japan; 10 Department of Hematology, Fukushima Medical University, Fukushima, Japan; 11 Department of Hematology, National Hospital Organization Sendai Medical Center, Sendai, Miyagi Japan; 12 Department of Hematology, Saiseikai Maebashi Hospital, Maebashi, Gunma Japan; 13 Department of Hematology and Oncology, Japanese Red Cross Society Narita Hospital, Narita, Chiba Japan; 14 Department of Hematology and Rheumatology, Faculty of Medicine, Kindai University, Sayama, Osaka Japan; 15 Department of Hematology, Kobe City Medical Center General Hospital, Kobe, Hyogo Japan; 16 Department of Hematology and Oncology, Okayama University Hospital, Okayama, Japan; 17 Department of Hematology, Sapporo Hokuyu Hospital, Sapporo, Hokkaido Japan; 18 Clinical Development Department, Nippon Shinyaku Co., Ltd, Kyoto, Japan; 19 Data Science Depertment, Nippon Shinyaku Co., Ltd, Kyoto, Japan; 20 Drug Metabolism and Pharmacokinetics Research Department, Nippon Shinyaku Co., Ltd, Kyoto, Japan; 21 Department of Hematology, Atomic Bomb Disease Institute, Nagasaki University, Nagasaki, Japan

**Keywords:** Acute myeloid leukemia, CPX-351, Cytarabine, Daunorubicin, Liposomal formulation

## Abstract

**Objectives:**

NS-87/CPX-351 is a dual-drug liposomal encapsulation of cytarabine and daunorubicin. NS-87/CPX-351 exerts antileukemic action by maintaining a synergistic molar ratio of cytarabine to daunorubicin of 5:1 within the liposome while in circulation. Patients with high-risk acute myeloid leukemia (AML), which includes therapy-related AML and AML with myelodysplasia-related changes (AML-MRC), have poorer outcomes than those with other AML.

**Methodology:**

This open-label phase 1/2 (P1/2) study was conducted in 47 Japanese patients aged 60–75 years with newly diagnosed high-risk AML to evaluate the pharmacokinetics, safety, and efficacy of NS-87/CPX-351.

**Results:**

In the 6 patients enrolled in the P1 portion, no dose-limiting toxicities (DLTs) were reported, and 100 units/m^2^ during the induction cycle was found to be acceptable. Cytarabine and daunorubicin had a long half-life in the terminal phase (32.8 and 28.7 h, respectively). In the 35 patients enrolled in the P2 portion, composite complete remission (CRc; defined as complete remission [CR] or CR with incomplete hematologic recovery [CRi]) was achieved in 60.0% (90% CI: 44.7–74.0) of the patients. Adverse events due to NS-87/CPX-351 were well tolerated.

**Outcomes:**

NS-87/CPX-351 can be considered as a frontline treatment option for Japanese patients with high-risk AML.

**Supplementary Information:**

The online version contains supplementary material available at 10.1007/s12185-024-03733-z.

## Introduction

Acute myeloid leukemia (AML) is primarily a disease of the elderly and is rapidly fatal [1]. AML can occur de novo, but it can also evolve from an antecedent hematologic disorder or as a complication of chemotherapy or radiation therapy, which together account for about 30% of all cases of AML [2, 3]. Patients with AML following previous myeloid malignancy (e.g., myelodysplastic syndromes (MDS) and myeloproliferative neoplasms) or therapy-related AML have poorer outcomes than those with de novo AML, and this is especially true for patients age 60 or older [3–6]. In these elderly patients, a poor performance status, a high incidence of comorbidities, and unfavorable genomic features can contribute to poor outcomes [4, 7]. Combination chemotherapy remains the standard of care for intensive AML induction therapy (e.g., a regimen of cytarabine for 7 days and daunorubicin for 3 days (7 + 3)), but patients with therapy-related or secondary AML respond poorly to this treatment [3, 5].

NS-87/CPX-351, known as Vyxeos Liposomal overseas, is a dual-drug liposomal encapsulation of cytarabine and daunorubicin. The notable clinical efficacy of NS-87/CPX-351 is achieved through maintenance of a synergistic molar ratio of cytarabine to daunorubicin of 5:1 within the liposome while in circulation in animal models [8]. The composition of the NS-87/CPX-351 liposome enables protection of both drugs from metabolism and elimination, overcoming the differences in pharmacokinetic profiles between the two compounds and reducing exposure of normal tissues to cytarabine and daunorubicin [9, 10].

In an international P3 study of patients with high-risk AML, which consists of therapy-related AML, AML with antecedent MDS or chronic myelomonocytic leukemia (CMML), or de novo AML with MDS-related karyotypes (the 301 study), NS-87/CPX-351 significantly improved median overall survival (OS) versus conventional chemotherapy with the 7 + 3 regimen (median, 9.56 vs. 5.95 months; hazard ratio (HR), 0.69; 95% confidence interval (CI), 0.52–0.90; one-sided p = 0.003) and the median survival after transplantation was also prolonged [11]. After 5 years of follow-up, the improved OS with NS-87/CPX-351 versus conventional chemotherapy was maintained [12]. The incidence of hematologic and non-hematologic adverse events in the 301 study was comparable between treatment arms. NS-87/CPX-351 was approved for the treatment of newly diagnosed therapy-related AML or myelodysplasia-related changes (AML-MRC) in the US as well as the EU.

In Japan, more effective therapies are also needed for this high-risk population. Thus, a P1/2 study was conducted to evaluate the pharmacokinetics, safety, and efficacy of NS-87/CPX-351 in elderly Japanese patients with therapy-related AML, AML with antecedent MDS or CMML, or de novo AML with MDS-related karyotypes. This study was registered with the Japan Registry of Clinical Trials (jRCT2080224810).

## Materials and methods

### Eligibility criteria

Eligible patients were age 60–75 years with newly diagnosed AML according to the WHO criteria published in 2017 [2]. Eligible patients were confirmed to have one of the following: therapy-related AML, AML with antecedent MDS or CMML, or de novo AML with MDS-related karyotypes. Other enrollment criteria included an Eastern Cooperative Oncology Group (ECOG) status of 0–2 [13]. Patients whose cardiac ejection fraction (EF) was more than 50% according to echocardiography or a multiple-gated acquisition scan within 28 days of the start of NS-87/CPX-351 were eligible.

The following patients were excluded: (1) patients who had previously received induction therapy for AML; (2) patients with acute promyelocytic leukemia (t(15;17)) or favorable cytogenetics, including t(8;21) or inv16; and (3) patients with prior cumulative anthracycline exposure of daunorubicin 368 mg/m^2^ or greater or its equivalent (218 mg/m^2^ for patients who received radiation therapy to the mediastinum). Details on the inclusion and exclusion criteria are provided in the Supplementary Material.

### Study oversight

The Institutional Review Board of each participating hospital approved the study protocol, and the study was conducted in accordance with the Good Clinical Practice for Drugs and the Declaration of Helsinki. All patients provided written informed consent before participation in the study.

### Study design and treatment

This open-label, single-arm study consisted of a P1 portion and P2 portion. In the P1 portion, pharmacokinetics and safety, including dose-limiting toxicity (DLT), were evaluated using the 3 + 3 design after the administration of one cycle of NS-87/CPX-351. In the first induction cycle, three eligible patients were administered a starting dose of 100 units/m^2^ NS-87/CPX-351 (100 mg/m^2^ cytarabine and 44 mg/m^2^ daunorubicin). If none of the three patients experienced a DLT, an additional three patients received the same dose. The P2 was planned to be initiated if none or only one of the six patients experienced a DLT.

**Fig. 1:**
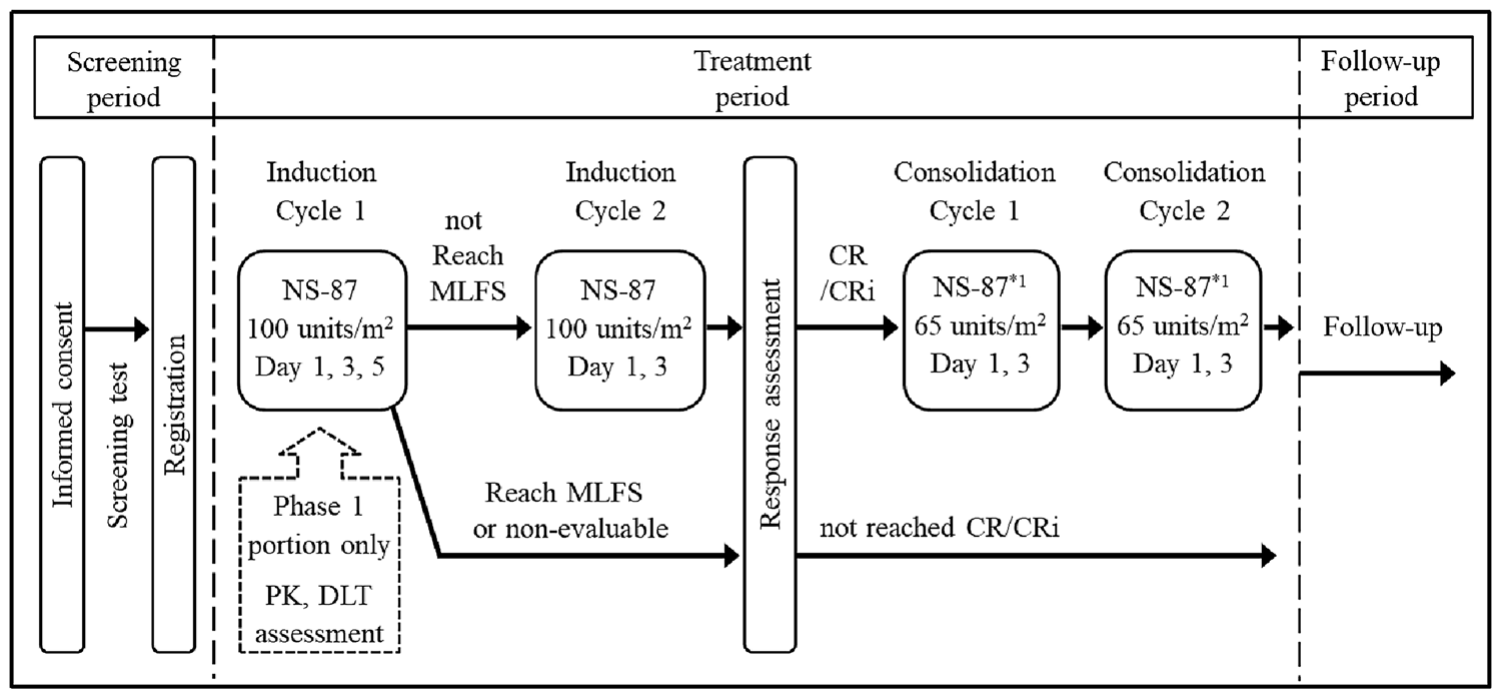
Study design and treatment. *CR* Complete Remission, *CRi* Complete Remission with Incomplete Blood Count Recovery, *PK* pharmacokinetics, *DLT* dose-limitting toxicity, *MLFS* morphologic leukemia-free state (bone marrow blasts <5% and absence of Auer rods and/or extramedullary disease). *1 Investigators treating patients with cumulative anthracycline exposure ≥500 mg/m^2^ had the option to receive an alternative consolidation regimen of intermediate-dose cytarabine, 1.5 g/m^2^ BID on Days 1, 3 and 5. Patients with less than 500 mg/m^2^ cumulative anthracycline exposure, including the study treatment, who have a >10% absolute decrease in LVEF to less than 50%, received the alternative consolidation regimen of intermediate-dose cytarabine as described above

In the P2 portion (including patients enrolled in the P1 portion), efficacy and safety were evaluated after a maximum of two induction cycles and two consolidation cycles (Fig. 1).

Patients could receive up to two cycles of induction chemotherapy to achieve complete remission (CR) or CR with incomplete neutrophil or platelet recovery (CRi) followed by up to two cycles of consolidation therapy in the treatment period. The initial NS-87/CPX-351 induction cycle consisted of 100 units/m^2^ administered intravenously as a 90-min infusion on Days 1, 3, and 5 during hospitalization.

A second induction cycle (at the same dose) was administered on Days 1 and 3 to patients who did not have hypoplastic marrow according to a bone marrow assessment on Day 15 in the first cycle. For patients in whom CR/CRi was achieved after induction, consolidation therapy consisted of up to two cycles of 65 units/m^2^ NS-87/CPX-351 (65 mg/m^2^ cytarabine and 29 mg/m^2^ daunorubicin) on Days 1 and 3. Investigators had the option of using an intermediate dose of cytarabine, depending on the left ventricular EF or anthracycline dosage, but no investigators chose it (Fig. 1). After completion or discontinuation of treatment, allogeneic hematopoietic cell transplantation (HCT) could be performed at the discretion of the investigator.

### End points and assessments

The primary efficacy end point was the rate of composite complete remission (CRc: CR + CRi; assessed by investigators according to the Revised International Working Group Criteria for AML) at the end of the induction cycles [14]. Secondary efficacy end points were overall survival (OS), event-free survival (EFS), relapse-free survival (RFS), a morphologic leukemia-free state (MLFS), and the rate of subjects transferred for HCT. OS was defined as the number of days from the start of treatment to the date of death; EFS was defined as the number of days from the start of treatment to persistent disease, relapse from CR or CRi, or death; RFS was defined as the number of days from first response (CR or CRi) to relapse or death. Cytogenetic risk was evaluated according to the NCCN guidelines for AML, version 1.2019.

Safety outcomes were adverse events (AEs), laboratory results, vital signs, cardiac functional tests, and physical findings. The severity of AEs was graded according to the National Cancer Institute Common Terminology Criteria for Adverse Events, version 5.0.

DLTs were assessed in cycle 1 of the P1 portion to decide which patients would transition to the P2 portion. The definition of DLTs is shown in the Supplementary Material.

### Pharmacokinetics

Pharmacokinetic samples to determine cytarabine and daunorubicin plasma concentrations or pharmacokinetic parameters were collected from 6 patients in the P1 portion on Days 1 and 5 of the first induction cycle: at 0.75 (or the midpoint of the infusion), 1.5 (at the end of infusion), 2, 4, 6, 8, or 24 h after the start of drug infusion (SOI), besides pre-dose on Day 1, and at 28, 48, 72, 96, 168, or 216 h post-SOI on Day 5. Copper pharmacokinetics were also monitored because the NS-87/CPX-351 formulation contains copper. Pharmacokinetics samples to determine copper serum concentrations or pharmacokinetics parameters were also obtained from the same number of patients. The serum samples for copper were collected on Day 1: pre-dose, 1.5, 2, 8, or 24 h after SOI, and on Day 5: pre-dose, 1.5, 2, 8, or 24, 96 or 216 h after SOI. The serum samples for copper were also collected on Day 1 of the second induction cycle and every consolidation cycle pre-dose, at the end of treatment (EOT), and 60 ± 10 days after EOT. Plasma concentrations of cytarabine and daunorubicin were determined using validated liquid chromatography and mass-spectrometry (LC–MS/MS; LC, Nexera X2 systems [Shimadzu, Kyoto, Japan]; MS/MS, API5000 [SCIEX, MA, USA]) after acidification to measure intra- and extra-liposomal concentrations. A validated analytical method using an inductively coupled plasma optical emission spectrometer (ICP-OES; SPECTRO Analytical Instruments GmbH, Kleve, Germany) was used to determine copper concentrations in serum. Pharmacokinetic parameters were determined using the concentrations obtained from total cytarabine or daunorubicin in plasma and copper in serum according to a non-compartmental pharmacokinetic analysis with Phoenix WinNonlin™ version 6.3 (Certara, NJ, USA).

### Statistical analysis

No formal sample size calculations were performed for P1 portion. Given the CRc rate of 47.7% for NS-87/CPX-351 and 33.3% for 7 + 3 in the 301 study, the anticipated results and the threshold for CRc rate were 50 and 30%, respectively. Enrollment of 35 patients to P2 portion was sufficient to detect 50% in CRc rate with at least 80.5% power and a one-sided significance level of 0.05. The date when the last patient completed the induction cycles served as the cutoff date for clinical data. Efficacy was to be evaluated with the data on the cutoff date and 1 year after the cutoff date. Safety was evaluated in all patients who received at least one NS-87/CPX-351 dose (safety analysis set [SAF]) with the data on the cutoff date. The efficacy analysis set was the full analysis set (FAS) consisting of all treated patients and excluding patients deemed not to meet the target disease criteria by an independent review committee (IRC).

The number and rate of patients in whom CRc was achieved were evaluated as the primary efficacy endpoint, which was defined as the best response observed across all time points during the induction cycles. The Clopper-Pearson interval was used to calculate the CIs for this endpoint. The distribution of time-to-event endpoints, including the median OS and its 90% CI, was estimated using the Kaplan–Meier method. Univariate logistic regression analysis tests, Cox proportional hazard regression analysis and univariate Cox analysis were performed to estimate 90% CIs, the p-value, and the odds ratio or the HR.

## Results

### Patient characteristics

**Fig. 2:**
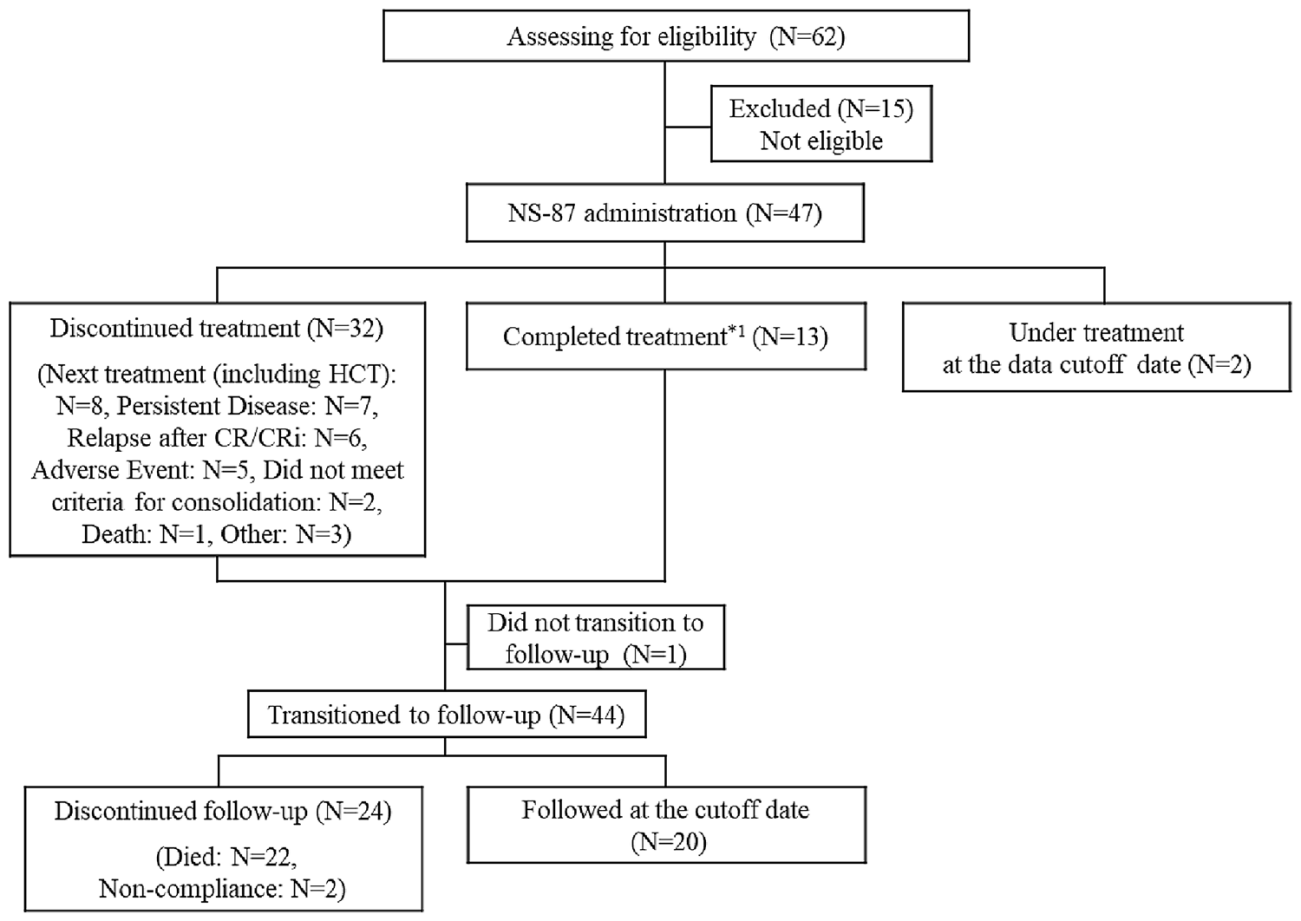
Deposition of patients. *1 Completed treatment is a patient group that completed the treatment (induction and consolidation cycles). *HCT* hematopoietic cell transplantation, *CR* complete remission, *CRi* CR with incomplete recovery

Patients were enrolled between August 2019 and October 2021 across 21 sites in Japan. Six patients were enrolled in the P1 portion and 56 in the P2 portion. A total of 6 patients in the P1 portion and 41 in the P2 portion received NS-87/CPX-351 treatment (Fig. 2). Of the 47 treated patients, seven were excluded from the FAS population. These patients were determined not to meet the criteria for AML according to the IRC. During the treatment period, 25 patients (53.2%) transitioned to the consolidation cycle and 13 (27.7%) completed the two pre-defined consolidation cycles. The most common reasons for discontinuation of NS-87/CPX-351 were transitioning to the next treatment, which included HCT (8 patients [25%]) during the NS-87/CPX-351 treatment period, and death (22 patients [92%]) during follow-up. Table 1 shows the baseline characteristics of patients enrolled in both portions of the study.

**Table 1:** Patient baseline characteristics

	Phase I portion (N = 6)	Phase II Portion (N = 41)	Overall (N = 47)
Sex n (%)			
Male	3 (50.0)	28 (68.3)	31 (66.0)
Female	3 (50.0)	13 (31.7)	16 (34.0)
Age, years			
Mean ± (SD)	67.7 (4.7)	67.9 (4.3)	67.8 (4.3)
Median	66.5	68.0	68.0
min, max	62.0, 75.0	60.0, 75.0	60.0, 75.0
≥70 yr	2 (33.3)	17 (41.5)	19 (40.4)
ECOG PS n (%)			
0	3 (50.0)	21 (51.2)	24 (51.1)
1	2 (33.3)	18 (43.9)	20 (42.6)
2	1 (16.7)	2 (4.9)	3 (6.4)
Type of AML n (%)			
Therapy-related AML	0 (0.0)	2 (4.9)	2 (4.3)
AML with antecedent MDS with previous HMAs	0 (0.0)	5 (12.2)	5 (10.6)
AML with antecedent MDS without previous HMAs	6 (100.0)	29 (70.7)	35 (74.5)
De novo AML with MDS karyotype	0 (0.0)	4 (9.8)	4 (8.5)
AML with antecedent CMML	0 (0.0)	1 (2.4)	1 (2.1)
Cytogenetic risk group n (%)			
Intermediate	2 (33.3)	21 (51.2)	23 (48.9)
Poor	4 (66.7)	20 (48.8)	24 (51.1)
Previous anthracycline exposure n (%)			
Yes	0 (0.0)	1 (2.4)	1 (2.1)
No	6 (100.0)	40 (97.6)	46 (97.9)
White blood cell count, × 10^3^/μL			
Mean ± (SD)	11.5 (15.6)	6.2 (10.3)	6.9 (11.0)
Median	2.2	2.5	2.5
min, max	1.0, 35.9	0.7, 54.9	0.7, 54.9
Platelet count, × 10^3^/μL			
Mean ± (SD)	114.8 (85.0)	90.6 (123.2)	93.7 (118.5)
Median	116.5	55.0	56.0
min, max	14.0, 248.0	10.0, 770.0	10.0, 770.0
Bone marrow blasts %			
Mean ± (SD)	29.3 (7.0)	40.6 (20.5)	39.1 (19.7)
Median	30.7	32.6	32.6
min, max	20.6, 36.4	20.0, 96.0	20.0, 96.0

The median age was 68 years, and 40.4% of the patients were over 70 years of age. Two patients (4.3%) had therapy-related AML, and 40 patients (85.1%) had AML with antecedent MDS. Of the patients diagnosed with AML with antecedent MDS, 5 (10.6%) had previously received a hypomethylating agent (HMA). Twenty-four patients (51.1%) had an ECOG rating of PS 0 and 24 (51.1%) had a poor cytogenetic risk.

### Pharmacokinetics

**Fig. 3:**
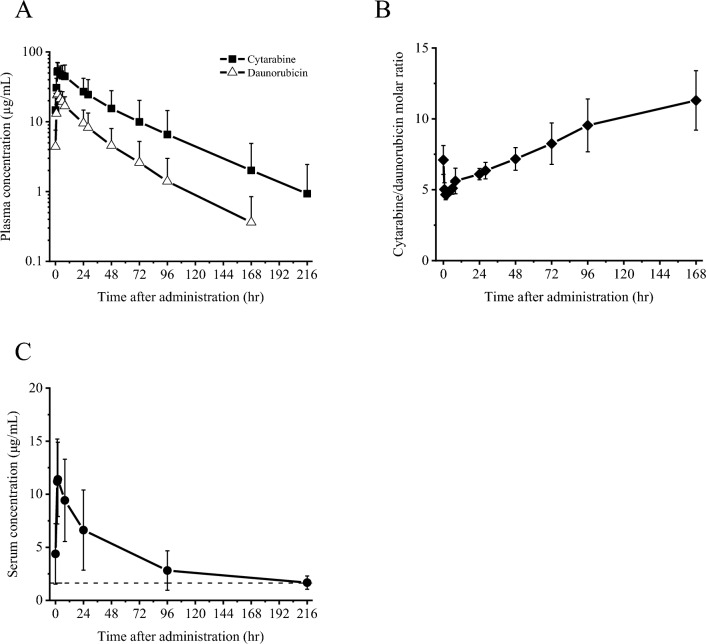
Pharmacokinetics. **A** Plasma concentration curves for cytarabine and daunorubicin after a 90-min intravenous infusion of NS-87/CPX-351 100 units/m^2^ on Day 5 (semi-log scale). Each plot represents the mean + SD of 6 subjects. **B** Molar ratio curve for cytarabine/daunorubicin after a 90-min intravenous infusion of NS-87/CPX-351 100 units/m^2^ on Day 5 (normal scale). Each plot represents the mean ± SD of 6 subjects. **C** Serum concentration curves for copper after a 90-min intravenous infusion of NS-87/CPX-351 100 units/m^2^ on Day 5 (normal scale). Each plot represents the mean ± SD of 6 subjects. The dotted line represents the upper 95% confidential interval of the mean baseline copper levels (1.63 μg/mL)

Summary pharmacokinetic parameters for total cytarabine, total daunorubicin, and copper on Day 5 are shown in Table 2. Cytarabine and daunorubicin had a long half-life in the terminal phase (t_1/2_) (32.8 and 28.7 h, respectively) and single-exponential, first-order elimination with limited α-phase distribution (Fig. 3).

**Table 2:** Pharmacokinetic parameters for cytarabine or daunorubicin in plasma and copper in serum after a 90-min intravenous infusion of NS-87/CPX-351 100 units/m^2^ on Day 5

Pharmacokinetic parameter	Cytarabine	Daunorubicin	Copper
T_max_ (hr)	1.99 (1.42–7.92)	1.98 (1.55–2.00)	1.77 (1.42–2.00)
C_max_ (µg/mL)	55.8 ± 18.1	25.0 ± 7.5	11.5 ± 3.7
AUC_0-tau_ (µg·hr/mL)	1430 ± 730	529 ± 237	352 ± 170
t_1/2_ (hr)	32.8 ± 11.8	28.7 ± 9.9	NA
V_z_ (L)	6.23 ± 2.44	6.20 ± 1.91	NA
CL_ss_ (L/hr)	0.154 ± 0.101	0.172 ± 0.101	NA
R_acc_C_max_	1.32 ± 0.32	1.14 ± 0.17	NA
R_acc_AUC_tau_	1.43 ± 0.33	1.27 ± 0.20	NA
T > Baseline (hr)	NA	NA	158 ± 66

n = 6, *NA* Not Applicable; Mean ± SD; except for t_max_, Median (Minimum–Maximum); t_max_

R_acc_C_max_ was calculated based on C_max_ and R_acc_AUC_tau_ from the AUC_0-48 h_ post-dose on Day 1

The molar ratio of cytarabine to daunorubicin was calculated based on total drug concentrations in plasma post-dose on Day 5. The mean molar ratio for the first 24 h after dosing ranged from 4.65 to 6.10 except for pre-dose (Fig. 3). Thus, the molar ratio of cytarabine to daunorubicin based on both drug concentrations was maintained at a synergistic ratio of around 5:1 for up to 24 h.

The copper concentration was temporarily elevated approximately sevenfold compared to the upper 95% CI for the mean baseline copper levels (1.63 µg/mL) following the end of infusion on Day 5; however, the mean time for the copper concentration to return to the baseline level was 158 h or about 6.5 days (Fig. 3).

### DLTs

All six patients in the P1 portion were included in the DLT evaluable population.

No DLTs were reported in these patients, and a dose of 100 units/m^2^ of NS-87/CPX-351 on Days 1, 3, and 5 during an induction cycle was acceptable.

### Safety

Overall, 47 patients were included in the SAF population (6 in the P1 portion and 41 in the P2 portion).

All patients experienced at least one AE, and 24 patients (51.1%) had serious AEs. The common AEs are summarized in Table 3. The most frequently reported hematologic AEs (frequency of ≥50% of patients) were febrile neutropenia (85.1%), thrombocytopenia (59.6%), and anemia (53.2%). Non-hematologic AEs with a frequency of ≥30% were a rash (42.6%), constipation (40.4%), pyrexia (38.3%), nausea (34.0%), and stomatitis (34.0%). The most frequently reported grade 3–5 AEs were febrile neutropenia (80.9%), thrombocytopenia (59.6%), anemia (51.1%), leukopenia (31.9%), and neutropenia (31.9%).

**Table 3:** Adverse events in patients administered NS-87/CPX-351

	All Grades (N = 47)	≥ Grade 3 (N = 47)
All adverse events	47 (100.0)	47 (100.0)
Hematologic adverse events^a^		
Febrile neutropenia	40 (85.1)	38 (80.9)
Anemia	25 (53.2)	24 (51.1)
Thrombocytopenia	28 (59.6)	28 (59.6)
Leukopenia	15 (31.9)	15 (31.9)
Neutropenia	15 (31.9)	15 (31.9)
Non-hematologic adverse events^a^		
Rash	20 (42.6)	2 (4.3)
Constipation	19 (40.4)	0 (0.0)
Pyrexia	18 (38.3)	5 (10.6)
Nausea	16 (34.0)	1 (2.1)
Stomatitis	16 (34.0)	1 (2.1)
Pneumonia	12 (25.5)	8 (17.0)
Insomnia	11 (23.4)	0 (0.0)
Hypokalemia	10 (21.3)	4 (8.5)
Decreased appetite	10 (21.3)	5 (10.6)
Serious adverse events^b^	24 (51.1)	24 (51.1)
Febrile neutropenia	11 (23.4)	11 (23.4)
Pneumonia	7 (14.9)	7 (14.9)
Pyrexia	4 (8.5)	4 (8.5)
Sepsis	2 (4.3)	2 (4.3)
Cellulitis	2 (4.3)	2 (4.3)

N (%); adverse events were coded using MedDRA Version22.0

^a^Adverse events of any grade that were reported in at least 20% of patients are listed

^b^Serious adverse events that were reported in at least 3% of patients are listed

By the data cutoff date, there had been 23 deaths (48.9%) overall. The most common primary cause of death was transformation to leukemia (16 patients, 34.0%). The causes of death due to AEs were pneumonia (2 patients), sepsis (1 patient), and cerebral hemorrhage (1 patient) (Supplementary Material).

Five patients experienced AEs that led to the discontinuation of treatment (pneumonia in 3 patients, cerebral haemorrhage in 1 patient, and a femoral neck fracture in 1 patient). All occurred within the first 20 days of treatment. Infusion of NS-87/CPX-351 was prolonged due to an infusion-related AE in 3 patients.

Nearly all patients (97.9%) were anthracycline-naïve, but anthracycline-induced cardiotoxicity was closely monitored. Cardiac AEs were experienced by 29.8% of patients and drug-related AEs were experienced by 12.8%. The only cardiac AE with a frequency of ≥10% was peripheral edema (10.6%) (Supplementary Material).

Almost all of the patients (97.9%) experienced at least 1 infection-related AE. AEs with a frequency of ≥10% were febrile neutropenia (85.1%), pyrexia (38.3%), and pneumonia (25.5%). Bleeding-related AEs with a frequency of ≥10% were disseminated intravascular coagulation and epistaxis (10.6% each).

### Efficacy

This section describes the efficacy in the P2 portion, which was evaluated as the primary objective.

#### Primary endpoint

In the 35 patients included in the FAS population, the CRc (CR + CRi) rate was 60.0% (90% CI: 44.7–74.0; 21/35 patients), which exceeded the null hypothesis rate of 30.0%. CR was achieved in 40.0% of patients and CRi in 20.0% (Table 4). The CRc rate and CR rate at the end of the first course of induction cycle was 45.0% (18/35 patients) and 30.0% (12/35), respectively. No patients with a CRi response were upgraded to CR during consolidation therapy.

**Table 4:** Treatment response and secondary endpoints

	All	Age		AML subtype					Cytogenetic risk group	
		60–69 years	70–75 years	Therapy-related AML	AML with antecedent MDS with previous HMAs	AML with antecedent MDS without previous HMAs	de novo AML with MDS karyotype	AML with antecedent CMML	Intermediate	Unfavorable
N	35	19	16	2	5	24	3	1	16	19
CR + CRi N(%)	21 (60.0)	12 (63.2)	9 (56.3)	2 (100.0)	2 (40.0)	15 (62.5)	2 (66.7)	0 (0.0)	10 (62.5)	11 (57.9)
CR N (%)	14 (40.0)	8 (42.1)	6 (37.5)	2 (100.0)	1 (20.0)	9 (37.5)	2 (66.7)	0 (0.0)	7 (43.8)	7 (36.8)
CRi N (%)	7 (20.0)	4 (21.1)	3 (18.8)	0 (0.0)	1 (20.0)	6 (25.0)	0 (0.0)	0 (0.0)	3 (18.8)	4 (21.1)
Median OS (months) (90% CI)	8.58 (6.77, 13.28)	8.25 (6.77, 13.28)	10.72 (6.58, NA.)	NA (4.54, NA.)	6.77 (0.59, NA.)	10.72 (6.71, NA.)	8.58 (3.16, 8.58)	9.40 (NA., NA.)	Not reached (6.71, NA.)	7.43 (6.58, 10.72)
Median EFS (months) (90% CI)	4.67 (2.04, 6.05)	4.67 (1.87, 7.04)	3.60 (1.81, NA)	2.47 (2.47, NA)	1.87 (0.53, NA)	5.33 (2.04, 10.7)	2.63 (0.69, NA)	0.72 (NA., NA)	5.10 (2.04, NA.)	4.47 (0.95, 6.05)
Median RFS (months) (90% CI)	8.88 (3.91, 12.1)	5.29 (2.73, NA)	8.88 (4.24, NA)	1.48 (1.48, NA)	NA	8.88 (4.24, NA)	2.09 (1.25, NA)	NA	11.2 (4.24, NA)	3.91 (2.73, NA)
Rate of MLFS achieved N (%)	N = 33 25 (75.8)	N = 19 14 (73.7)	N = 14 11 (78.6)	N = 2 2 (100.0)	N = 5 3 (60.0)	N = 22 18 (81.8)	N = 3 2 (66.7)	N = 1 0 (0.0)	N = 14 12 (85.7)	N = 19 13 (68.4)
Rates of HCT N (%)	11 (31.4)	10 (52.6)	1 (6.3)	2 (100.0)	0 (0.0)	7 (29.2)	1 (33.3)	1 (100.0)	5 (31.3)	6 (31.6)

*CR* Complete Remission, *CRi* Complete Remission with Incomplete Blood Count Recovery, *OS* Overall Survival, *EES* Event-free Survival, *RFS* Relapse-free Survival, *MLFS* Morphologic Leukemia-free State, *HCT* Hematopoietic Cell Transplantation, *NA* Not applicable

The prespecified subgroup analyses revealed no significant differences in the CRc rate depending on age, AML subtype, or cytogenetic risk categories, with the exception of therapy-related AML and AML with antecedent CMML, where only a few patients were evaluated (Table 4). CRc was achieved in 56.3% of the patients over 70 years of age and was comparable to the CRc rate (63.2%) in patients younger than age 70.

#### Secondary endpoints

The median duration of follow-up was 204 days (range: 18–681 days). The median OS was 8.58 months (90% CI: 6.77–13.28) (Table 4). The Kaplan–Meier estimate of the 1-year OS was 36.58% (90% CI: 20.12–53.21).

The median OS in patients 60–69 years of age was 8.25 months (90% CI: 6.77–13.28), and that in patients over 70 years of age was 10.72 months (90% CI: 6.58–not reached [NR]). In patients with an intermediate cytogenetic risk (n = 16), the median OS was not reached (90% CI: 6.71 to not estimable [NE]), whereas the median OS was relatively poor (7.43 months; 90% CI: 6.58–10.72) in patients with a poor cytogenetic risk (n = 19). OS data as of one year after the cutoff date are shown in Supplementary Material. The median post-HCT OS at one year after the cutoff date was 3.32 months (90% CI: 2.50, 10.03) (Supplementary Material). The causes of post-HCT death were primary disease (n = 7) and post-transplant pneumonia (n = 1).

The median EFS was 4.67 months (90% CI: 2.04–6.05), and the median RFS was 8.88 months (90% CI: 3.91–12.13) (Table 4). Eleven of 35 patients (31.4%) underwent HCT. Of the 11 patients who underwent HCT, 10 were younger than age 70, while one was 71. MLFS was achieved in 75.8% of patients (25/33 patients). The results of exploratory univariate logistic and Cox regression analysis of baseline prognostic factors associated with CRc and OS are shown in the Supplementary Material. In univariate Cox regression analysis, the bone marrow blast count was found to be associated with OS in the FAS population, with a p-value of 0.081.

The median time up to neutrophil (≥1,000/μL) and platelet (≥100,000/μL) recovery in patients in whom CR was achieved after initial induction chemotherapy was 35.0 days (25th percentile [Q1]: 28.0; 75th percentile [Q3]: 40.5) and 35.0 days (Q1: 28.0, Q3: 39.0) respectively. In patients in whom either CR or CRi was achieved, the median time to neutrophil and platelet recovery were 36.0 days (Q1: 27.0, Q3: 43.0) and 37.0 days (Q1: 29.0, Q3: 55.0), respectively. Changes in platelet and neutrophil counts in patients in whom CR was achieved after initial induction chemotherapy are shown in the Supplementary Material.

## Discussion

This P1/2 study was designed to confirm the pharmacokinetics, efficacy, and safety of NS-87/CPX-351 in Japanese adult patients with high-risk AML.

The patient characteristics in this study are similar to those in the 301 study; for example, 40.4% of patients in this study were over 70 years of age compared to 36% in the 301 study.

The median bone marrow blast count at diagnosis (32.6% in this study and 35.0% in the 301 study) and the percentage of patients with a poor cytogenetic risk (51.1 and 50.3%, respectively) were also similar in this study and the 301 study, suggesting that patients with a similar prognosis had been enrolled.

The mean volume of distribution (Vz) during the terminal elimination phase following intravenous administration of both drugs was about 6 L, corresponding to less than 5% of the volume of distribution for non-liposomal cytarabine (138 L) or daunorubicin (1364 L) [15]. Since the volume of distribution for both drugs was approximately the same as the blood volume (5.2–7.5 L), the extent of NS-87/CPX-351 distribution was considered to be mostly restricted to the blood. The pharmacokinetic parameters for NS-87/CPX-351 in Japanese patients were similar to those seen in Caucasian patients with AML [16]. In the Japanese P1/2 study and the study overseas, the molar ratio of cytarabine to daunorubicin was maintained at the targeted ratio of 5:1 in plasma for 24 h after administration, which could have contributed to the potent antileukemic action of NS-87/CPX-351. NS-87/CPX-351 contains 100 mg copper gluconate per vial, of which 14% is elemental copper. Upon its administration, the serum copper concentrations transiently increased and then decreased to near baseline after the infusion completed. However, there is a need to exercise caution when administering NS-87/CPX-351 to patients with Wilson’s disease or other copper metabolic diseases.

The persistence of NS-87/CPX-351 liposomes in the plasma may also prolong the exposure of the bone marrow to the drug and increase the cytotoxic activity and killing of leukemic cells relative to the free drugs [11, 12], but it could also be related to the longer time neutrophils and platelets took to recover. In the 301 study, the median time to neutrophil and platelet recovery in patients in whom CR was achieved after initial induction chemotherapy was approximately 1 week longer with NS-87/CPX-351 than with the 7 + 3 regimen [11]. Since the median time to neutrophil and platelet recovery in patients who received NS-87/CPX-351 in this study was similar to that in the 301 study, the risk of infection or bleeding events should be kept in mind.

The safety profile of NS-87/CPX-351 is consistent with the known adverse reaction profiles of cytarabine and daunorubicin. No DLTs were reported, and the profiles and frequencies of AEs were similar to those reported in the 301 study. The most common grade ≥3 AEs were hematologic events, and these events were manageable with appropriate monitoring and intervention, including with the use of antibiotic or antifungal therapy. Anthracyclines are known to cause cardiac toxicities, but no problematic cardiotoxicity was observed in this study, just as none was observed in the 301 study. However, cardiac function should be closely monitored, and evaluation with an electrocardiogram or via echocardiography before and after administration is recommended, especially in patients with risk factors for increased cardiac toxicity, as well as 7 + 3 treatment.

The rate of early (30- and 60-day) mortality was lower with NS-87/CPX-351 (5.9 and 13.7%) than with 7 + 3 treatment (10.6 and 21.2%) in the 301 study [11]. The 30- and 60-day mortality rates in the current study were 6.4 and 8.5%, respectively, which were on par with those in the 301 study. The lower early mortality rates observed may be due to the preferential uptake of NS-87/CPX-351 by leukemic cells [17]. Early mortality is a major risk of administering intensive chemotherapy to patients with AML, and the risk is particularly high in the elderly. Reducing early mortality is considered to be a major benefit for patients with AML receiving intensive chemotherapy, and especially elderly patients.

The CRc rate in Japanese patients with high-risk AML also was high (60%) in this study; which exceeded the rate reported in the 301 study (47.7%). There were few patients in each group and all patients were eligible for intensive chemotherapy despite their age. These are factors to bear in mind, but a high rate of remission was achieved in almost all subpopulations, including patients over the age of 70. These results indicate that NS-87/CPX-351 may be effective in very elderly patients with AML.

The median survival time in this study was similar to that in the 301 study. Ongoing and future analyses need to be performed to more comprehensively evaluate the efficacy of NS-87/CPX-351. The 5-year follow-up from the 301 study revealed a prolonged OS, so a further survival benefit may be expected in Japanese patients with a longer follow-up. Numerous studies have reported reduced survival in patients with AML-MRC defined by cytogenetics or a history of MDS, compared to patients with AML with multilineage dysplasia but without other MRC [14, 18, 19]. In this P1/2 study, the median OS in the AML dysplasia group (single or multilineage dysplasia only, n = 8) was longer compared to AML-MRC subgroups defined by cytogenetics (n = 19) or a history of MDS (n = 14) (NR vs. 8.25 months vs. 6.77 months, respectively). Although post hoc analyses evaluated the effect of NS-87/CPX-351 treatment on OS in multiple subgroups, there were very few patients in these subgroups, limiting the ability to draw conclusions based on these data. In the 301 study, genetic analysis indicated that patients with two of the most common mutations (DNMT3A and TET2) had a longer median OS when receiving NS-87/CPX-351 versus conventional chemotherapy [20]. The genetic characteristics of Japanese patients enrolled in this study and the association between gene mutations and outcomes will be evaluated.

In the 301 study, the median EFS was significantly prolonged with NS-87/CPX-351 versus 7 + 3 (2.53 vs. 1.31 months; HR: 0.74; 95% CI: 0.58–0.96; two-sided p-value: 0.021). The median EFS in this P1/2 study was 4.67 months (90% CI: 2.04–6.05), so NS-87/CPX-351 may be expected to be efficacious in Japanese patients as well.

In the 301 study, patients given NS-87/CPX-351 were more likely to proceed to HCT than were those treated with conventional chemotherapy with the 7 + 3 regimen (34.0 vs. 25.0%). Moreover, the improved post-HCT survival was observed in patients randomly assigned to NS-87/CPX-351 compared to the 7 + 3 regimen [21]. In this Japanese P1/2 study, 31.4% of patients received HCT, which was similar to the percentage in the 301 study. The median OS landmarked from the allo-HCT date was 10.3 months in all patients (P1 and P2 portion) on the cutoff date. The rate of HCT will change from these cutoff data because one additional patient underwent HCT after the data cutoff date. Although in the P1 and P2 portion, the percentage of patients over 70 years of age who underwent HCT was lower (5.6% [1/18]) than that of patients under 70 years of age (50.0% [11/22]). Achieving remission is essential for patients who do not undergo HCT. In the 301 study, the median OS for patients in whom CR or CRi was achieved but who did not undergo HCT (NS-87/CPX-351, n = 33; 7 + 3, n = 28) was numerically longer with NS-87/CPX-351 than with the 7 + 3 regimen (14.72 vs. 7.59 months; HR: 0.57; 95% CI: 0.31, 1.03) [22]. In this P1/2 study, the median OS in patients who were classified in the same category was 12.10 months.

In conclusion, this P1/2 study found that NS-87/CPX-351 tends to result in a high rate of remission and that its safety and pharmacokinetic profiles are comparable to those in the clinical study overseas. The results of this study suggest a clinical benefit with NS-87/CPX-351 in Japanese adult patients with high-risk AML.

## Limitation

A limitation of this study was the small number of patients who met the study criteria. Additional studies need to be conducted to confirm these findings. The diagnosis of high-risk AML may vary among physicians, so patients should be carefully evaluated for this drug combination.

### Supplementary Information

Below is the link to the electronic supplementary material.

Supplementary file1 (DOCX 217 KB)

## Declarations

### Data availability

All data generated or analyzed during this study are included in this published article and its supplementary information files.
