# Supplementary material for: A phase 1/2 study of NS-87/CPX-351 (cytarabine and daunorubicin liposome) in Japanese patients with high-risk acute myeloid leukemia
Source: Int J Hematol. 2024 Mar 26;119(6):647–59. doi: 10.1007/s12185-024-03733-z (PMC11136735; doi:10.1007/s12185-024-03733-z)
Supplement: Supplementary file 1 — Supplementary file1 (DOCX 217 KB) [file 12185_2024_3733_MOESM1_ESM.docx]

**Supplementary Material**

# **Title**

**A phase 1/2 study of NS-87/CPX-351 (cytarabine and daunorubicin liposome) in Japanese patients with high-risk acute myeloid leukemia**

# **Author names and affiliations**

Kensuke Usuki, MD, PhD^1^, Toshihiro Miyamoto, MD, PhD^2^, Takuji Yamauchi, MD, PhD^3^, Kiyoshi Ando, MD, PhD^4, 5^, Yoshiaki Ogawa, MD PhD ^4^, Masahiro Onozawa, MD, PhD^6^, Takahiro Yamauchi, MD, PhD^7^, Hitoshi Kiyoi, MD, PhD^8^, Akira Yokota, MD^9^, Takayuki Ikezoe, MD, PhD^10^, Yuna Katsuoka, MD, PhD^11^, Satoru Takada, MD, PhD^12^, Nobuyuki Aotsuka, MD, PhD^13^, Yasuyoshi Morita, MD, PhD^14^, Takayuki Ishikawa, MD, PhD^15^, Noboru Asada, MD, PhD^16^, Shuichi Ota, MD, PhD^17^, Atsushi Dohi^18^, Kensaku Morimoto^19^, Shunji Imai, PhD^20^, Umi Kishimoto^18^, Koichi Akashi, MD, PhD^3^, Yasushi Miyazaki, MD, PhD^21^ and the Study Group for NS-87/CPX-351^22^

# **Corresponding author**

Full name: Kensuke Usuki

Address: 5-9-22 Higashi-Gotanda, Shinagawa-ku, Tokyo 141-8625, Japan

E-mail address: kensuke.usuki@gmail.com

**Inclusion and exclusion criteria**

[Inclusion criteria]

Patients who met all of the following criteria and provided written informed consent

1. Japanese patients aged 60–75 years at the time of signing the informed consent form
2. Patients diagnosed with AML according to WHO criteria published in 2017.
3. Patients with AML corresponding to one of the following.

- Therapy-related AML

Requires prior cytotoxic therapy for an unrelated disease:

・alkylating agents

・ionizing radiation therapy: large fields including active bone marrow

・topoisomerase II inhibitors

・other: antimetabolites, antitubulin agents

- AML with a history of MDS

Requires any of the following:

・Previous confirmed history of MDS.

・Bone marrow evidence of dysplasia present in ≥10% of cells in one or more myeloid lineages or ≥10% dysplastic megakaryotypes.

・Unequivocal dysplasia in <10% of cell in one or more myeloid cell lines with clonal abnormalities characteristic of MDS (one of the following).

Clonal abnormalities: Unbalanced changes: +8*, -7 or del(7q), -5 or del(5q); del(20q)*, -Y*, i(17q) or t(17p), -13 or del(13q), del(11q), del(12p) or t(12p), del(9q), idic(X)(q13) Balanced changes: t(11;16)(q23;p13.3); t(3;21)(q26.2;q22.1); t(1;3)(p36.3;q21.2); t(2;11)(p21;q23); inv(3)(q21q26.2), t(6:9)(p23;q34)

*If the sole cytogenetic abnormality, also requires morphologic criteria with dysplasia present in ≥10% of cells in one or more myeloid lineages or ≥10% dysplastic megakaryotypes; all other clonal abnormalities are sufficient for a presumptive diagnosis

- *De novo* AML with karyotypic abnormalities characteristic of MDS

Requires any of the following:

・Complex karyotype (defined as 3 or more chromosomal abnormalities).

・Unbalanced: -7 or del(7q); -5 or del(5q); i(17q) or t(17p); -13 or del(13q); del(11q); del(12p) or t(12p); del(9q); idic(X)(q13).

・Balanced: t(11;16)(q23;p13.3); t(3;21)(q26.2;q22.1); t(1;3)(p36.3;q21.2); t(2;11)(p21;q23), t(5;12)(q33;p12); t(5;7)(q33;q11.2); t(5;17)(q33;p13); t(5;10)(q33;q21); t(3;5)(q25;q34)

- AML with a history of CMML

Requires a history of CMML, which requires the following (at diagnosis of CMML):

・Peripheral blood monocytosis >1000/μL

・Absence of Philadelphia chromosome or BCR-ABL1 fusion gene

・In the presence of eosinophilia, absence of rearrangements of PDGFRA or PDGFRB

・Presence of dysplasia in one or more myeloid lineages

・If myelodysplasia is absent/minimal, CMML may still be diagnosed if the above requirements are met and there is either:

・the presence of an acquired clonal cytogenetic or molecular genetic abnormality in hematopoietic cells or

・the persistence of monocytosis for ≥ 3 months and all other causes of monocytosis have been excluded

・There are fewer than 20% blasts (myeloblasts, monoblasts, or promonocytes) in peripheral blood and bone marrow.

1. Eastern Cooperative Oncology Group (ECOG) performance status of 0, 1, or 2
2. Patients whose laboratory results meet the following criteria within 3 days before the start of the study drug:

- Serum creatinine < 2.0 mg/dL;
- Serum bilirubin < 2.0 mg/dL (patients with Gilbert's syndrome should be judged by a specialist);
- Serum aspartate aminotransferase (AST) and alanine aminotransferase (ALT) < 3.0 times the upper limit of normal (ULN) (patients whose liver enzymes exceed ULN in relation to AML should be judged by a specialist);

1. Patients whose cardiac ejection fraction ≥ 50% according to echocardiography or a multiple-gated acquisition scan (MUGA) within 28 days prior to the start of the study drug
2. Patients who have agreed to use appropriate contraception (for example, contraceptive devices or contraceptive drugs) during the treatment period and for 6 months after administration.
3. Patients providing written informed consent
4. Patients who were deemed by a physician to be able to survive for more than 12 weeks after the start of treatment

[Exclusion criteria]

Patients who met any of the following criteria were excluded from the study.

- 1. Patients with a history of MPN (defined as a history of essential thrombocytosis or polycythemia vera, or idiopathic myelofibrosis except for CMML) or combined MDS/MPN prior to the diagnosis of AML
  2. Patients with acute promyelocytic leukemia [t(15;17)] or favorable cytogenetics, including t(8;21) or inv16.
  3. Patients with clinical evidence of active CNS leukemia.
  4. Patients with active (uncontrolled, metastatic) second malignancies.

Patients who have remained in remission according to imaging or tumor marker tests without recurrence for at least 6 months after completion of cytotoxic therapy can be enrolled (maintenance therapy with hormonal agents after remission, etc., is not counted as cytotoxic therapy).

- 1. Patients who previously received induction therapy for AML.

Hydroxyurea for disease control can be permitted until 24 hours before the start of the study treatment. Patients previously treated with a hypomethylating agent (HMA), cytarabine (AraC) alone (>1 g/m^2^/day), AraC plus an anthracycline or HSCT, etc. for AML are also excluded.

- 1. Patients who received any therapy for MDS within two weeks of the first dose of the study drug.

Adverse reactions associated with prior MDS treatment must have recovered to Grade 1 or less based on CTCAE v5.0 prior to start of the study treatment.

- 1. Patients who underwent any surgery (excluding local surgery) or radiation therapy within four weeks of the first dose of the study drug.
  2. Patients with prior cumulative anthracycline exposure of greater than 368 mg/m^2^ (218 mg/m^2^ for patients who received radiation therapy to the mediastinum) daunorubicin or the equivalent.
  3. Patients with any serious medical condition, laboratory abnormality, or psychiatric illness that would prevent the obtaining of informed consent.
  4. Patients with myocardial impairment due to any cause (e.g., cardiomyopathy, ischemic heart disease, significant valvular dysfunction, hypertensive heart disease, and congestive heart failure) resulting in heart failure according to the New York Heart Association Criteria (Class III or IV staging).
  5. Patients with an active or uncontrolled infection.

Patients with an infection for which they are receiving treatment (antibiotics, antifungals, or antivirals) can be enrolled but must be afebrile and hemodynamically stable for more than 72 hours.

- 1. Patients with current evidence of an invasive fungal infection (patients with a suspected infection must have a subsequent negative culture to be eligible) or known HIV.
  2. Patients with current evidence of an active hepatitis B or C infection (with rising transaminase values).
  3. Patients with hypersensitivity to cytarabine, daunorubicin or liposomal products.
  4. Patients with a history of Wilson’s disease or other copper metabolism disorder.
  5. Patients who received any treatment with another investigational drug or unapproved drug within 28 days of the first dose of the study drug.
  6. Pregnant women, women suspected of being pregnant, and lactating women.

Female patients should undergo a pregnancy test during the screening phase. However, this test is not required for menopausal patients who have been in menopause for more than one year since their last period without any other medical reason or for female patients who cannot become pregnant due to a hysterectomy or ovary removal. Interruption of breastfeeding will not allow inclusion in this study.

- 1. Patients who were deemed to be inappropriate for the study by the investigator (subinvestigator).

**Definition of DLTs**

DLT was defined using CTCAE version 4.0 for toxicity occurring during the first cycle of therapy only.

| DLT was defined as any grade 3 or 4 treatment-related non-hematologic toxicity occurring during the first cycle of therapy with the following exceptions:   - - Nausea or vomiting: Only grade 3 or 4 nausea and vomiting that was refractory to appropriate anti-emetic therapy was considered a DLT.   - Dysphagia, esophagitis, mucositis, and/or diarrhea: Only grade 3 events (lasting more than 7 days) or any grade 4 event were considered DLTs.   - Febrile neutropenia and documented infection: Only grade 4 febrile neutropenia and a documented infection are considered DLTs. |
| --- |
| Dose-limiting hematologic toxicity was defined as a bone marrow and peripheral blood examination at Day 56 or until the start of next treatment cycle showing the following findings:   - - Meeting the criteria for CR or CRi according to the Revised International Working Group Criteria for AML AND   - Persistently hypocellular marrow (<20% cellularity) AND   - Peripheral blood:   ・ANC< 500 AND/OR  ・Platelet count <10,000 or persistent platelet transfusion dependence |

**Tables**

| Factor | Factor level | n/N (%) | Hazard ratio  (90% CI) | Pairwise  p-value | Overall  p-value |
| --- | --- | --- | --- | --- | --- |
| Age | 60-69 | 19 / 35 ( 54.3) | 1.03 ( 0.52, 2.03) | 0.939 | 0.939 |
|  | 70-75 | 16 / 35 ( 45.7) |  |  |  |
| ECOG PS | 0 | 19 / 35 ( 54.3) | >99 (<0.00, n/a ) | 0.992 | 0.546 |
|  | 1 | 15 / 35 ( 42.9) | >99 (<0.00, n/a ) | 0.992 |  |
|  | 2 | 1 / 35  ( 2.9) |  |  |  |
| WBC category (10^3^/μL) | < 20 | 33 / 35 ( 94.3) | 1.54 ( 0.29, 8.31) | 0.672 | 0.672 |
|  | >= 20 | 2 / 35  ( 5.7) |  |  |  |
| Platelet category (10^3^/μL) | <= 50 | 17 / 35 ( 48.6) | 1.13 ( 0.58, 2.23) | 0.760 | 0.760 |
|  | > 50 | 18 / 35 ( 51.4) |  |  |  |
| Hemoglobin category (g/dL) | <= 9 | 25 / 35 ( 71.4) | 1.55 ( 0.70, 3.40) | 0.362 | 0.362 |
|  | > 9 | 10 / 35 ( 28.6) |  |  |  |
| Bone marrow blast count (%) | 20-40 | 20 / 35 ( 57.1) | 3.01 ( 1.19, 7.61) | 0.051 | 0.081 |
|  | >40 - 60 | 7 / 35  ( 20.0) | 1.29 ( 0.40, 4.17) | 0.717 |  |
|  | >60 | 8 / 35  ( 22.9) |  |  |  |

**Table S1 Summary of univariate Cox proportional hazard regression for OS**

N: number of patients with available data for the specified factor.

n: number of patients described by the specified factor.

p-value: two-sided p-value from a Wald chi-square test.

Pairwise p-value: p-values for levels within each factor.

Overall p-value: p-values for each factor.

**Table S2 Summary of univariate logistic regression for the response (CR or CRi) rate**

| Factor | Factor level | n/N (%) | Odds ratio  (90% CI) | Pairwise  p-value | Overall  p-value |
| --- | --- | --- | --- | --- | --- |
| Age | 60-69 | 12 / 19 ( 63.2) | 1.33  ( 0.43, 4.17) | 0.678 | 0.678 |
|  | 70-75 | 9 / 16 ( 56.3) |  |  |  |
| ECOG PS | 0 | 11 / 19 ( 57.9) | <0.00  (<0.00, >99) | 0.979 | 0.992 |
|  | 1 | 9 / 15 ( 60.0) | <0.00  (<0.00, >99) | 0.980 |  |
|  | 2 | 1 / 1 ( 100.0) |  |  |  |
| WBC category (10^3^/μL) | < 20 | 19 / 33 ( 57.6) | <0.00  (<0.00, >99) | 0.971 | 0.971 |
|  | >= 20 | 2 / 2 ( 100.0) |  |  |  |
| Platelet category (10^3^/μL) | <= 50 | 11 / 17 ( 64.7) | 1.47  ( 0.47, 4.60) | 0.581 | 0.581 |
|  | > 50 | 10 / 18 ( 55.6) |  |  |  |
| Hemoglobin category (g/dL) | <= 9 | 16 / 25 ( 64.0) | 1.78  ( 0.51, 6.18) | 0.447 | 0.447 |
|  | > 9 | 5 / 10 ( 50.0) |  |  |  |
| Bone marrow blast count (%) | 20-40 | 11 / 20 ( 55.0) | <0.00  (<0.00, >99) | 0.964 | 0.500 |
|  | >40 - 60 | 2 / 7 ( 28.6) | <0.00  (<0.00, >99) | 0.954 |  |
|  | >60 | 8 / 8 ( 100.0) |  |  |  |

N: number of patients with available data for the specified factor.

n: number of patients described by the specified factor.

p-value: two-sided p-value from a Wald chi-square test.

Pairwise p-value: p-values for levels within each factor.

Overall p-value: p-values for each factor.

**Table S3 OS data as of one year after the data cutoff**

|  | Phase I portion  n (%) | Phase II portion  n (%) | Overall  n (%) |
| --- | --- | --- | --- |
| Overall | 5 | 35 | 40 |
| Death n (%) | 3 ( 60.0) | 24 ( 68.6) | 27 (67.5) |
| Censored n (%) | 2 ( 40.0) | 11 ( 31.4) | 13 (32.5) |
| Median (months)  (90% CI) | 12.1  (4.11, 12.10) | 9.40  ( 6.77, 14.96) | 9.40  (6.77, 13.28) |
| 1-year OS (%)  (90% CI) | 60.0  (19.10, 85.42) | 41.20  (27.24, 54.64) | 42.94  (29.58, 55.62) |

**Table S4 Post-HCT OS data at one year after the data cutoff**

|  | Phase I portion  n (%) | Phase II portion  n (%) | Overall  n (%) |
| --- | --- | --- | --- |
| Overall | 1 | 11 | 12 |
| Death n (%) | 0 ( 0.0) | 8 ( 72.7) | 8 (66.7) |
| Censored n (%) | 1 ( 100.0) | 3 ( 27.3) | 4 (33.3) |
| Median (months)  (90% CI) | NA.  (NA., NA.) | 3.32  ( 2.50, 10.03) | 3.57  (2.50, NA.) |
| 1-year OS (%)  (90% CI) | 100.0  (100.00, 100.00) | 27.27  (8.86, 49.83) | 31.25  (11.20, 53.90) |

**Table S5 Adverse events that led to discontinuation of NS-87/CPX-351 treatment or death**

|  | | N=47 |
| --- | --- | --- |
| Adverse events that led to discontinuation of treatment | | 5 (10.6) |
|  | Pneumonia | 3 (6.4) |
|  | Femoral neck fracture | 1 (2.1) |
|  | Cerebral haemorrhage | 1 (2.1) |
| Adverse events that led to death | | 4 (8.5) |
|  | Pneumonia | 2 (4.3) |
|  | Sepsis | 1 (2.1) |
|  | Cerebral haemorrhage | 1 (2.1) |

N (%); Adverse events were coded using MedDRA Version22.0

**Table S6 Cardiac adverse events**

|  | | N=47 |
| --- | --- | --- |
| Cardiac adverse events | | 14 (29.8) |
|  | Edema peripheral | 5 (10.6) |
|  | Edema | 4 (8.5) |
|  | Atrial fibrillation | 2 (4.3) |
|  | Cardiac tamponade | 1 (2.1) |
|  | Cardiac failure congestive | 1 (2.1) |
|  | Cardiac failure | 1 (2.1) |
|  | Sinus bradycardia | 1 (2.1) |
|  | Left ventricular dysfunction | 1 (2.1) |
|  | Ejection fraction decreased | 1 (2.1) |
|  | Pulmonary oedema | 1 (2.1) |
|  | Peripheral swelling | 1 (2.1) |
|  | Blood creatine phosphokinase increased | 1 (2.1) |

N (%); adverse events were coded using MedDRA Version22.0

# **Figures**


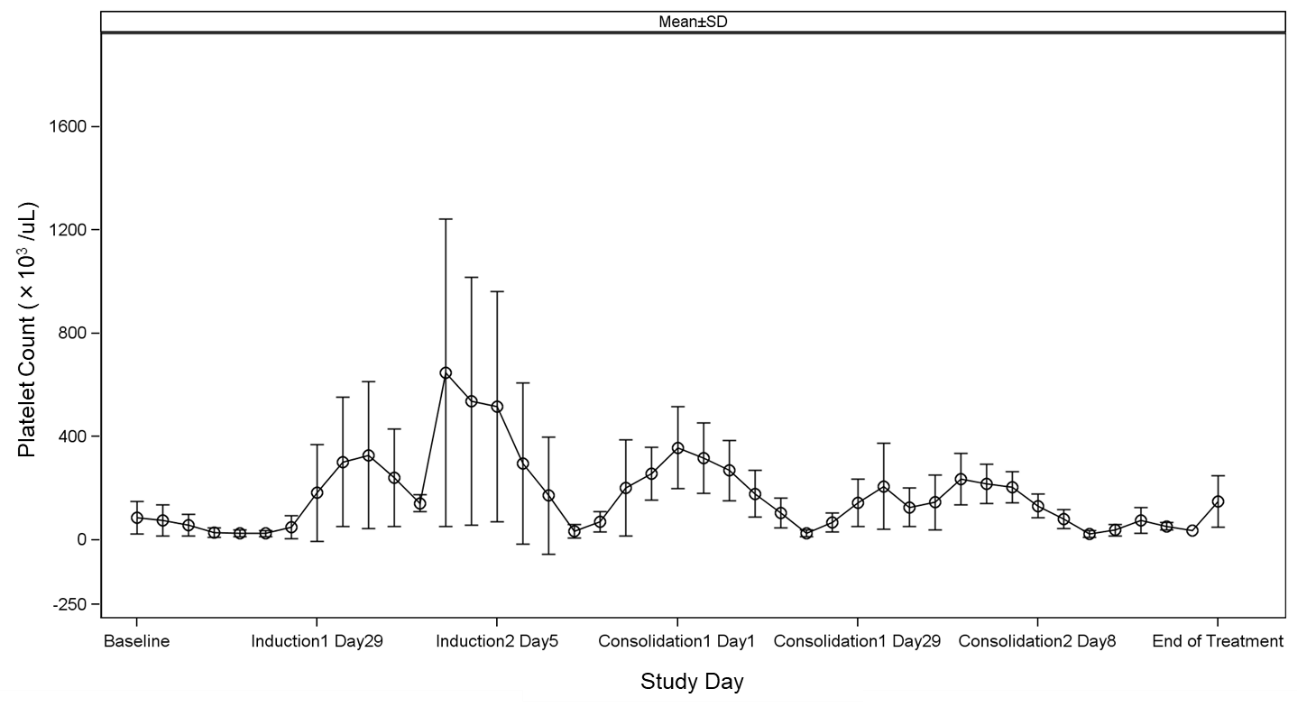
A


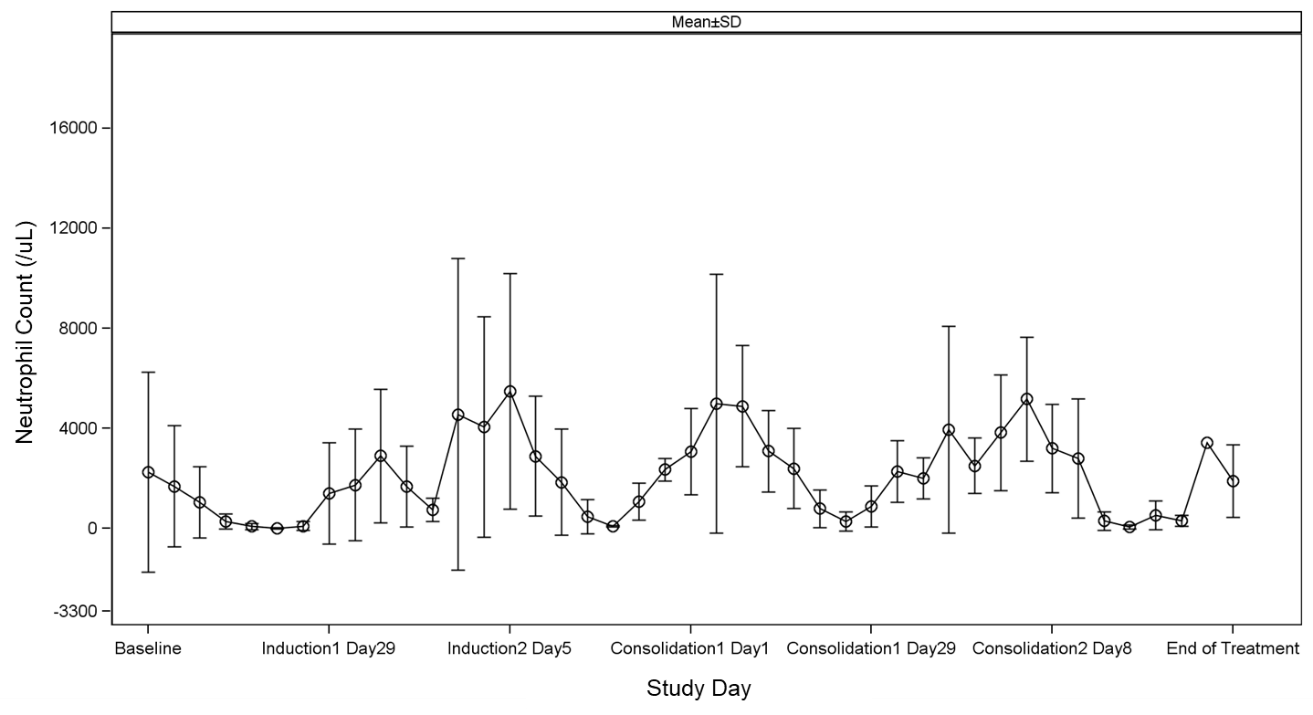
B

**Figure S1 Changes in platelet and neutrophil counts in patients in whom CR was achieved after induction cycles**

(A) Mean platelet counts from the baseline (pre-dose) to the end of NS-87 treatment. Each plot represents the mean ± SD of 14 subjects.

(B) Mean neutrophil counts from the baseline (pre-dose) to the end of NS-87 treatment. Each plot represents the mean ± SD of 14 subjects.

# **Study group**

These were the affiliations of the study group for NS-87/CPX-351 at the time of the study.

Junya Kuroda, Division of Hematology and Oncology, Kyoto Prefectural University of Medicine

Hiroatsu Iida, Department of Hematology, National Hospital Organization Nagoya Medical Center

Naohiro Sekiguchi, Department of Hematology, National Hospital Organization Disaster Medical Center

Katsuto Takenaka, Department of Hematology, Clinical Immunology and Infectious Diseases, Ehime University Graduate School of Medicine

Toshiro Kawakita, Department of Hematology, National Hospital Organization Kumamoto Medical Center

Kazunori Imada, Department of Hematology, Japanese Red Cross Osaka Hospital

Takahiro Suzuki, Department of Hematology, Kitasato University School of Medicine

Shuichi Miyawaki, Department of Hematology, Tokyo Metropolitan Otsuka Hospital

Noriko Usui, Department of Clinical Oncology and Hematology, The Jikei University School of Medicine

Norio Asou, Department of Hematology, International Medical Center, Saitama Medical University

Masakazu Muta, Clinical Laboratory, National Hospital Organization Kyusyu Cancer Center

Kazuto Tsuruda, Department of Laboratory Medicine, Nagasaki University Hospital

Masafumi Taniwaki, Center for Molecular Diagnostics and Therapeutics, Kyoto Prefectural University of Medicine

Masatoshi Fujita, Department of Cardiology, Uji Hospital

Hideki Makishima, Department of Pathology and Tumor Biology, Kyoto University

Yoko Nakanishi, Clinical Development Dept., Nippon Shinyaku Co., Ltd., Kyoto, Kyoto, Japan

Masaya Tajima, Clinical Development Dept., Nippon Shinyaku Co., Ltd., Kyoto, Kyoto, Japan

Yutaka Masutomi, Clinical Development Dept., Nippon Shinyaku Co., Ltd., Kyoto, Kyoto, Japan

Masahiro Chiba, Clinical Development Dept., Nippon Shinyaku Co., Ltd., Kyoto, Kyoto, Japan

Mayuna Hokazomo, Clinical Development Dept., Nippon Shinyaku Co., Ltd., Kyoto, Kyoto, Japan

Shihomi Hirooka, Clinical Development Dept., Nippon Shinyaku Co., Ltd., Kyoto, Kyoto, Japan

Taisuke Mikasa, Clinical Development Dept., Nippon Shinyaku Co., Ltd., Kyoto, Kyoto, Japan

Moemi Okamoto, Clinical Development Dept., Nippon Shinyaku Co., Ltd., Kyoto, Kyoto, Japan

Akitaka Kawase, Clinical Development Dept., Nippon Shinyaku Co., Ltd., Kyoto, Kyoto, Japan

Akane Yamada, Clinical Development Dept., Nippon Shinyaku Co., Ltd., Kyoto, Kyoto, Japan

Yuto Shimizu, Clinical Development Dept., Nippon Shinyaku Co., Ltd., Kyoto, Kyoto, Japan

Kento Isogaya, Data Science Dept., Nippon Shinyaku Co., Ltd., Kyoto, Kyoto, Japan

Tomohiko Ichikawa, Pharmacokinetics and Safety Assessment Dept., Nippon Shinyaku Co., Ltd., Kyoto, Kyoto, Japan
